# Refractory Gout Attack

**DOI:** 10.1155/2012/657694

**Published:** 2012-11-25

**Authors:** Simone Fargetti, Claudia Goldenstein-Schainberg, Andressa Silva Abreu, Ricardo Fuller

**Affiliations:** Rheumatology Division, Hospital das Clínicas da Faculdade de Medicina, Universidade de São Paulo (USP), Avenida Doutor Arnaldo 455, 01246-903 São Paulo, SP, Brazil

## Abstract

Refractory gout attack is an uncommon problem, since gout flares are usually self-limited. This clinical condition is characterized by serum uric acid higher than 6 mg/Dl or continuous manifestations of recurrent flares, chronic arthritis, and increased tophi. We report in this paper a 69-year-old man with a polyarticular and protracted gout attack, despite usual treatment and low urate sera levels. In order to manage this problem, we reviewed gout pathophysiology and developed a therapeutic solution based on benzbromarone pharmacokinetics. We also review herein new options for gout treatment that could be used in similar cases.

## 1. Introduction 

Gout is a multisystemic inflammatory disease characterized by recurrent flares of intense arthritis. The usual attack of gout arthritis normally has short duration, affects one or few joints, and exhibits good response to nonsteroidal anti-inflammatory drugs, colchicine and corticosteroids. Most cases occur as a consequence of the reduction of urinary excretion of uric acid, and therefore, can be managed with uricosuric medications such as benzbromarone. This drug has a rapid onset of action and reaches peak serum levels within four hours following ingestion, although its effect persists for at least one day, and, thus, benzbromarone is usually prescribed once daily. We report, in this paper, the case of a man with a longstanding established diagnosis of gout who suffered a polyarticular continuous attack of arthritis lasting 3, 5 months despite treatment with benzbromarone. Of note, clinical improvement with rapidly resolution of the acute attack occurred after the fractionation of benzbromarone dosage. We also reviewed the mechanisms of articular inflammatory process in gout and new options for gout treatment that could be used in similar cases. 

## 2. Case Report 

**Figure 1:**
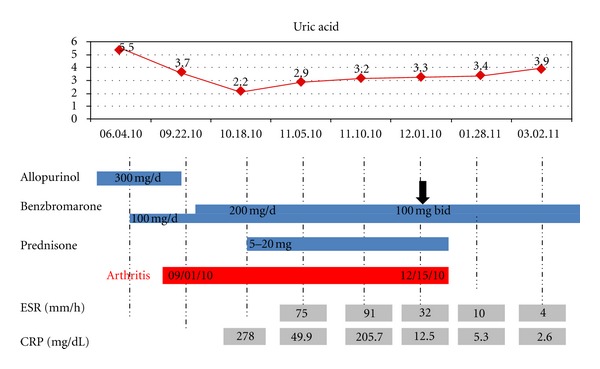
Graphic demonstrating reported gout patient outcomes: uric acid (red line), drugs (blue bar), laboratory tests (gray bar), and arthritis duration (red bar).

A 69-year-old Caucasian man was admitted to our rheumatology unit with a history of chronic tophaceous gout since 1980. At that time, he had monoarticular episodes that progressed over the years TO difficult to control polyarticular attacks. He was regularly taking allopurinol 300 mg daily and colchicine, nonsteroidal anti-inflammatory drugs, or intramuscular corticosteroids during the acute attacks. His gout flares lasted 5 to 14 days and occurred on average one time per month. Initial laboratory tests (May 2010) revealed serum uric acid 5.5 mg/dL, 24-hour uric acid urinary excretion level 194 mg/24 h, and uric acid clearance 2.4 mL/min. At this point, benzbromarone 100 mg daily was introduced, in order to increase urinary uric acid excretion. Despite good adherence, on September 2010, the patient developed a new episode of acute polyarticular gouty arthritis, affecting wrists, knees, ankles, right elbow, and first metatarsophalangeal joints. Serum uric acid was 3.7 mg/dL, 24-hour urine uric acid level 317 mg/24 h, uric acid clearance 5.9 mL/min, and creatinine 1.9 mg/dL. Prednisone 20 mg/day was initiated aiming to control the flare, and benzbromarone dose increased to 200 mg daily, Serum urate levels decreased to 2.2 mg/dL, but polyarthritis persisted, and the patient remained corticosteroid dependent (Figure 1) with an exuberant arthritis of the right knee. Due to the fact that the patient had evidence of high inflammatory activity, systemic and localized infectious and neoplastic screening was performed, with no evidence for any significant change or septic complication.

In fact, arthrocentesis of this joint withdrew 40 mL of low-viscosity and turbid liquid and revealed negatively birefringent needle-shaped crystals using polarized microscopy and 19.000 cells/mm^3^, predominantly polymorphonuclears (90%). All synovial fluid cultures (aerobic, anaerobic, for fungi and mycobacteria) were negative and without neoplastic cells. Plain X-rays of feet showed subchondral cysts and typical gout bone erosions in the proximal interphalangeal joints bilaterally. 

On November 2010, two and a half months after the onset of the gouty attack, the patient still persisted with significant polyarthritis associated to elevated erythrocyte sedimentation rate (ESR) of 75 mm and C-reactive protein (CRP) of 278 mg/dL, even in the presence of low serum levels of uric acid (Figure 1). At this point, based on benzbromarone pharmacokinetics, we decided to split the dose of this uricosuric agent to 100 mg twice a day (Figure 1). 

With this approach, the patient rapidly developed progressive improvement of his polyarthritis. As a consequence, within one month the patient was able to walk again normally, with complete resolution of arthritis and normalization of CRP and ESR sera levels (Figure 1), allowing prednisone tapering and withdrawal. Currently, our patient is on good control of gout, following this very long and intense flare that lasted three and a half months. No new onset of attacks has developed for more than 18 months, and our patient remains free of corticosteroids and using benzbromarone twice daily. 

## 3. Discussion 

The patient illustrated herein demonstrates some particular features: a prolonged polyarticular gout episode, refractory to conventional treatment, and accompanied by normal uric acid levels during disease progression.

Few data is published in the literature regarding refractory gout. It is estimated that difficult-to-treat gout affects about 1% of the overall gout patients in the United States [1]. Fels and Sundy have defined refractory gout as ongoing clinical manifestations (recurrent flares, chronic arthritis, and increasing tophi) or the failure to reduce serum uric acid below 6 mg/dL [2]. The same authors point out some reasons to explain refractory disease, such as delayed or insufficient dosing of urate lowering therapy and poor patient compliance or intolerance to medication [2]. In the case of our patient, urate lowering therapy was initiated early, in a proper dose and with good compliance. Therefore, we believe that particular factors related to gout pathophysiology and/or particular metabolic mechanisms may have played an essential role in this patient, resulting in a difficult to treat attack, despite adequate therapy. Acute polyarticular gout may occur in 15% to 40% of patients, especially women [3, 4]. These individuals tend to have long disease duration, insidious, ascending, and asymmetrical attacks, similar to our patient [5, 6], who presented not only a refractory and polyarticular arthritis, but also normal uric acid levels. It is well known that 22% to 33% of gout patients present serum uric acid levels below 7 mg/dL during an acute attack [7–9]. It usually occurs due to the use of urate-lowering drugs throughout the flare and to the uricosuric effects of adrenocorticotropic hormone (ACTH) that is released during the attack. Besides, local factors of involved joints may promote crystal formation and a sustained attack, despite normal serum uric acid [7]. 

Physiologically, during day time, the synovial fluid is able to maintain a constant concentration of intra-articular monosodium urate (MSU) crystals. At night, there is an articular dehydration attributed to joint rest, causing the passage of interstitial fluid to the circulatory system. In this process, oversaturation of synovial fluid with MSU crystals occurs, leading to nocturnal onset of attacks. It is possible that factors causing increased articular concentration of crystals, despite normal serum uric acid, may cause protracted arthritis. Another hypothesis to explain refractory attacks could be the low serum uric acid levels promoting tophi dissolution due to the concentration gradient. As a consequence, MSU crystals would spread to interstitium, causing areas of higher concentration and new crystals formation, perpetuating the inflammatory state.

In order to assess the best treatment in the normouricemic and refractory attack, it is necessary to consider the pathophysiology of gout. The initial phase is the formation of MSU crystals in a synovial fluid supersaturated with MSU [10]. These crystals are phagocytosed by monocytes, leading to the production of proinflammatory cytokines, such as TNF-*α*, interleukin-8 and the activation of NALP3 inflammasome, resulting in increased production of interleukin-1*β* (IL-1), the most important cytokine in the process [11]. Next step is the amplification phase, in which there is activation of adhesion proteins and leukocyte recruitment to the synovial tissue by IL-1, opsonization of MSU crystals by IgG and complement. This process facilitates MSU phagocytosis by monocytes and neutrophils, resulting in tissue damage [10, 12]. Finally, the resolution phase spontaneously develops after 48 hours of the attack onset, in which some mechanisms are proposed, such as binding of inhibitory molecules to the crystals, notably apolipoprotein B and apolipoprotein E, that prevent their recognition by phagocytes. There is also enhanced expression of peroxisome proliferator activated *γ*-receptor (PPAR-*γ*), that stimulates the apoptosis of neutrophils and macrophages, and of transforming growth factor-*β* (TGF-*β*), an antiinflammatory cytokine that inhibits leukocyte infiltration and endothelial activation [10, 12, 13]. All these mechanisms may fail in patients with refractory arthritis, preventing the self-limited natural course of acute gout. 

It is important to emphasize again that the management of refractory gout must involve not only the control of symptoms, but mainly the hyperuricemia. Current treatment of the acute flare consists in use of nonsteroidal anti-inflammatory drugs, colchicine and corticosteroids. Lately, there are new drugs being studied, the IL-1 inhibitors. For hyperuricemia, traditional drugs such as allopurinol and uricosuric drugs (benzbromarone) are mandatory, and more recently, the introduction of new drugs, such as febuxostat, an inhibitor of xanthine oxidase, and the modified uricases offer new options for our therapeutic arsenal.

It is known that allopurinol generally is used in low doses, below 300 mg, achieving low uric acid levels (below 5 mg/dL) in only 26% of patients. However, the increase to 600 mg may improve the success rates to 78% [14]. In our case, the patient presented chronic renal failure (estimated creatinine clearance of 35 mL/min); therefore, it was opted to stop allopurinol and increase benzbromarone to 200 mg, drug that can be used in renal failure [15], and show equal or better success rates to decrease serum urate, from 78% to 92% of patients attaining the target of 5 mg/dL [14, 16]. 

The role of biological therapy in the management of gout has also been proposed. Anti-TNF agents, a cytokine that plays some role in the disease, have been used in few case reports [17, 18], although experimental studies in rats with gout have not shown any inflammation inhibition of the acute response in this disease [19]. The IL-1 receptor antagonist anakinra, approved for rheumatoid arthritis, was studied in 10 patients with acute and polyarticular gout, resolving the attack in 9 patients without adverse effects [19]. Two new biologics directed against interleukin 1 are currently being studied in acute gout; they have recently been approved for the treatment of cryopyrin-associated periodic syndrome (CAPS), autoinflammatory diseases characterized by marked activation of NALP3 inflammasome. Rilonacept, a soluble IL-1 receptor-Fc fusion protein, was evaluated in 10 refractory gout patients, resulting in decrease of pain and C-reactive protein. A good safety profile was shown; however, no improvement in the number of affected joints was demonstrated [20]. Canakinumab is an IL-1 fully human monoclonal antibody investigated in a phase 2 study that involved 200 patients with acute gout. An intramuscular injection of 150 mg canakinumab was superior to triamcinolone acetonide, and no toxicity or infections were observed [21]. 

Our patient had a refractory gout flare for several weeks, despite low serum uric acid levels and optimized anti-inflammatory treatment. A possible explanation for this could be serum oscillation of uric acid levels during the day leading and/or allied to urate hyperconcentration in the involved joints. Therefore, we intended to maintain low and stable urate levels based on benzbromarone pharmacokinetics. This uricosuric agent is usually prescribed once daily because its metabolites have a half-life of 17–20 h [22, 23]. However, the peak action of this medication occurs within 4 hours following intake. Therefore, there is asymmetric activity during the day. For that reason, we changed the usual approach and prescribed the same dose of benzbromarone, although in split doses, that is, in two daily doses, in order to achieve a stable low serum uric acid levels through both day and night times, eliminating MSU crystals synovial hyperconcentration and activating the resolution phase. 

In conclusion and based on the experience and knowledge that we acquired with this patient, we propose that adequate management of refractory gout flares requires some particular and singular steps: (a) optimization of anti-inflammatory therapy allowing patient comfort; (b) initiation of uricosuric therapy even during the ongoing attack; and (c) more importantly maintaining low and stable serum urate levels. Furthermore, we present a new proposal for the treatment of refractory gout attacks based on benzbromarone pharmacokinetics.
